# Research on surface defect detection algorithm of pipeline weld based on YOLOv7

**DOI:** 10.1038/s41598-024-52451-3

**Published:** 2024-01-22

**Authors:** Xiangqian Xu, Xing Li

**Affiliations:** School of Material Science and Engineering, Xi’an Shiyou University, No. 18, East Section of Electronic Second Road, Xi’an, 710065 Shaanxi China

**Keywords:** Engineering, Materials science

## Abstract

Aiming at the problems of low target detection accuracy and high leakage rate of the current traditional weld surface defect detection methods and existing detection models, an improved YOLOv7 pipeline weld surface defect detection model is proposed to improve detection results. In the improved model, a Le-HorBlock module is designed, and it is introduced into the back of fourth CBS module of the backbone network, which preserves the characteristics of high-order information by realizing second-order spatial interaction, thus enhancing the ability of the network to extract features in weld defect images. The coordinate attention (CoordAtt) block is introduced to enhance the representation ability of target features, suppress interference. The CIoU loss function in YOLOv7 network model is replaced by the SIoU, so as to optimize the loss function, reduce the freedom of the loss function, and accelerate convergence. And a new large-scale pipeline weld surface defect dataset containing 2000 images of pipeline welds with weld defects is used in the proposed model. In the experimental comparison, the improved YOLOv7 network model has greatly improved the missed detection rate compared with the original network. The experimental results show that the improved YOLOv7 network model mAP@80.5 can reach 78.6%, which is 15.9% higher than the original model, and the detection effect is better than the original network and other classical target detection networks.

## Introduction

With the continuous improvement of industrialization, welding technology^1^ is widely used in various key fields such as ship transportation, petroleum industry, national defense science, and equipment manufacturing^2^. In the oil field, the welding quality of oil pipelines will directly affect the performance and life of the welded structure. During welding, the welded parts will be affected by the production equipment and process and the experience of the staff, and different degrees and quantities of defects will be formed at the welding site^3^. The generation of these defects is inevitable. If they are ignored, it will affect the performance of the entire pipeline, especially in some application scenarios such as oilfield operations. In severe cases, it may bring unpredictable safety accidents.

To ensure the quality of welded pipes, efficient and accurate defect detection of the weld seam is required. At present, industrial defect detection is mainly based on manual detection^4^ and machine vision detection. Manual detection has problems such as low detection efficiency, low detection accuracy and high false detection rate^5^. There are a large number of methods in machine vision detection. The traditional target detection algorithm^6,7^ is based on sliding window traversal to select the region, and then performs feature extraction and classification. However, the region selection algorithm has high computational complexity. Manual extraction of features is more complex, and the detection accuracy is limited. In recent years, the research and application of deep learning convolutional neural networks (CNN) have developed rapidly and achieved remarkable results in the field of computer vision^8^. In 2015, Girshick proposed Fast R-CNN^9^, which inputted the entire image into the neural network, generated the region of interest (ROI) by selecting the search, and uses the ROI pooling layer to obtain the corresponding features of each ROI region. In the same year, Ren et al. proposed Faster R-CNN^10^, which automatically generated ROI regions through region proposal network (RPN) instead of selective search, further improving the detection speed of the model. In 2015, Joseph et al. proposed the YOLO algorithm^11^, which greatly improved the detection speed and accuracy of the model. Subsequently, scholars have proposed YOLOv2-7 and other algorithms^12–16^, which further improved the accuracy and detection speed of the model.

With the emergence of one-stage detection algorithm YOLO, it has been gradually applied to the surface detection of pipeline weld defects with extremely fast detection speed and high accuracy. Melakhsou et al.^17^ applied the YOLOv3 algorithm to the weld quality inspection task to improve the efficiency of weld defect detection. Based on YOLOv3, Kou et al.^18^ used anchor-free method to improve the speed of the model, and designed dense convolution blocks to extract richer feature information, so as to improve the accuracy and robustness of the model. Han et al.^19^ improved the detection effect by rotating the prediction box and the rotation detector, but only for remote sensing scenes. Zhu et al.^20^ effectively improved the detection performance of the network for small-size targets by increasing the target detection layer, using the transformer prediction head, and integrating the CBAM attention module^21^, but it was easy to cause missed detection in dense cases. Although YOLO-z^22^ has achieved a good fusion of shallow and middle features by replacing PAFPN with Bi-FPN and expanding Neck layer, it is not suitable for scenes with large target size changes.

Compared with the above algorithm, the YOLOv7 model proposed by Wang et al.^16^ has faster speed and higher accuracy on the COCO dataset. The detector can greatly improve detection accuracy without increasing the inference cost. In the range of 5FPS to 160FPS, its detection speed and accuracy exceed all known target detectors. It has shown excellent performance in defect detection^16^, and the model can be used in practical engineering applications to meet the real-time requirements of pipeline weld surface defect detection. However, there are few researches on the application of YOLOv7 algorithm in the field of pipeline weld surface defect detection, and the accuracy of pipeline weld surface defect detection needs to be improved. In addition, the detection accuracy is also easily affected by the complex background and small defect targets of the pipeline weld image.

Aiming at the problems in the above-mentioned pipeline weld surface defect detection, an improved YOLOv7 pipeline weld surface defect detection algorithm is proposed. The algorithm is based on the current advanced single-stage target detector YOLOv7. Multiple improvements of network structure and loss function are made to adapt to more difficult defect detection tasks.

The main contributions of this study are:

1. According to different pipeline weld defects, a new pipeline weld data set is prepared, which can meet the detection of common pipeline weld surface defects, especially weld pore defects.
2. A Le-HorBlock module is designed and added to the YOLOv7 network, which realizes second-order spatial interaction through gated convolution and recursive design to enhance the extraction of important features in the target by the network.
3. The CoordAtt mechanism is introduced into the backbone network. By embedding target position information into channel attention, the performance of weld defect feature extraction is improved.
4. In order to solve the problem of unstable convergence of loss function in the process of target detection, the loss function is improved. The minimum angle formed by the connection between the center point of the two anchor frames and the horizontal direction is included in the loss calculation, which improves the convergence speed.

## Related work

### Development of defect detection technology

With the rapid development of object detection research and the continuous improvement of computer computing power, scholars have shifted from traditional detection methods to deep learning methods for welding surface defects^23^. Fu et al.^24^ proposed a CNN model for acquiring deep-level semantic features of targets, and combined it with multi-receptive fields to realize rapid and accurate classification of steel surface defects. Han et al.^25^ proposed a new detection method based on encoder-decoder residual networks (EDR-Net). In the coding stage, the fully convolutional neural network (FCN) was used to extract defect features, and the convergence of the model was accelerated by combining attention mechanisms. As the mainstream target detection framework in two-stage detection algorithm, Faster R-CNN is widely applied to weld surface defects. Aiming at the problems of large noise and low recognition accuracy of welding defect data set, Zhi et al.^26^ designed a parallel serial multi-scale feature information fusion mechanism and channel domain attention strategy, and constructed a deep learning network model based on Faster R-CNN. The recognition accuracy of defect types could reach more than 90%. Chen et al.^27^ proposed a new Faster R-CNN network model based on the improved ResNet50 to solve the problem of multi-scale target detection environment and poor performance of small target detection in existing algorithms.

Compared with the traditional methods, the above methods have improved the defect detection performance, but there are still problems of low overall detection accuracy and slow detection speed. Therefore, a new and improved YOLOv7 method is proposed in this paper, which improves the missed detection rate and accuracy of the model by improving its network structure, increasing attention mechanism and optimizing the loss function.

### The network of YOLOv7

**Figure 1:**
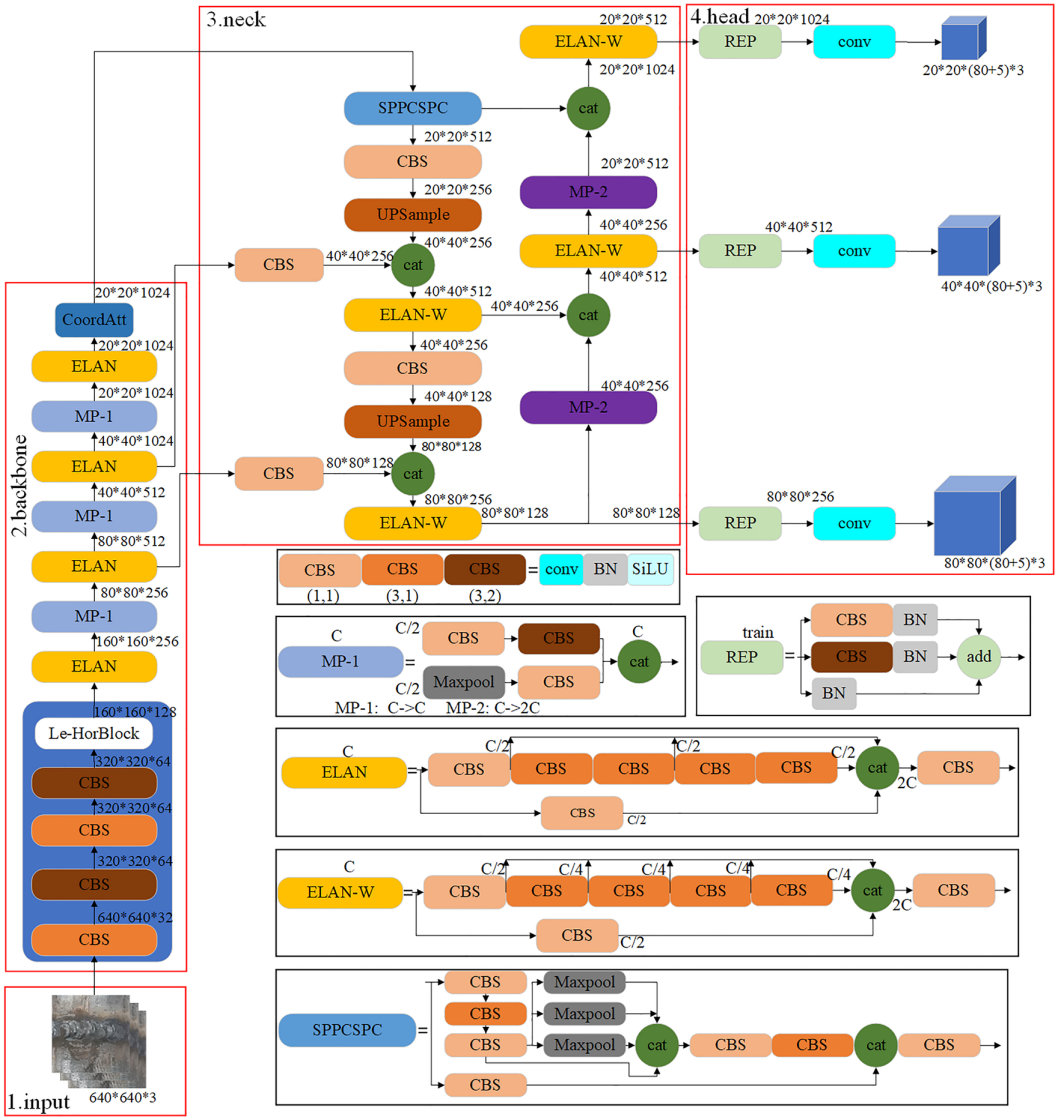
Structure of the improved YOLOv7 network.

The structure of YOLOv7 network^16^ is divided into four main parts, which are input, backbone network, neck network and head network, as shown in the circled red rectangle in Fig. 1. After a series of operations such as data enhancement of the input section, the image is resized to 640 × 640, and then fed into the backbone network to extract the image features. Subsequently, the extracted features are fused in the neck network to output three different sizes of features, large, medium and small. Finally, the fused features are sent to the detection head, and the results are output after detection.

The backbone network part of YOLOv7 consists of CBS, ELAN and MP-1 module. Among them, the CBS module consists of regular convolution, batch normalization, and activation functions, where batch normalization can speed up the training of the model and prevent the gradient from disappearing. The ELAN module consists of several CBS modules, which can learn the data effectively by controlling the gradient path and its deep network structure, and enable the model to converge better. The advantage of this design is that it avoids the infinite superposition of computational units and does not make the original gradient path steady state destroyed. The MP-1 module consists of the Maxpool and CBS modules, in which the Maxpool operation expands the perceptual field of the current feature layer and then fuses it with the feature information after normal convolution processing to further improve the feature extraction capability of the network. The structural diagrams of the above three modules are circled with black rectangles in Fig. 1.

The neck network mainly consists of a path aggregation network (PAN)^28^ and a feature pyramid network (FPN)^29^, which is used to fuse the features extracted from the backbone network to obtain richer target features. As can be seen from the last part of the backbone in Fig. 1, after passing through the SPPCSP module, the 32-fold downsampled feature maps produced by the backbone network are reduced from 1024 to 512 channels, and then the feature maps are fused through the PAN-FPN structure, and three feature maps with different sizes are output, namely 20 × 20, 40 × 40 and 80 × 80, which are used to detect small targets, medium targets and large targets respectively. Small feature maps can provide deep semantic information, while large feature maps contain a lot of fine-grained information. Finally, the network outputs the prediction results through the Rep and conv modules^30^. Therefore, YOLOv7 can not only predict at different scales, but also fully learn the semantics of feature maps at different scales during the prediction process.

### Attention mechanism

Attention mechanisms have been widely used in deep learning research in recent years^31^. For the target detection task, numerous studies have shown that the addition of the attention module in the network can improve the representational capability of the network model^32^ and effectively reduce the interference of invalid targets, thus improving the detection of the target of attention and achieving the goal of improving the overall detection effect of the network model.

Attentional mechanisms can generally be divided into channel attentional mechanisms, spatial attentional mechanisms, and a combination of both attentional mechanisms. The more typical ones are the squeeze and excitation attention module (SE), which consists of squeeze and excitation operations^33^, and the convolutional block attention module (CBAM), which consists of spatial attention module and channel attention module^34^. However, the SE attention mechanism only considers the internal channel information, ignoring the importance of the target location information. CBAM only introduces the location information through the global pool on the channel, and can only capture local information, but can't obtain remote dependent information. Therefore, CoordAtt mechanism is introduced which embeds position information into channel attention.

### Loss function

In the target detection network, target localization is done by the bounding box regression module. The IoU loss function is mainly used for the prediction box to be close to the ground truth box so as to improve the localization effect^35^. But for the case that the prediction box and the ground truth box do not intersect, the IoU loss function is difficult to converge the localization.

The GIoU^36^ proposed in 2019 obtained the weight of the prediction box and the ground truth box in the closed region by introducing the smallest box that can surround the prediction box and the ground truth box. However, the GIoU loss function becomes the IoU loss function when the prediction box and the ground truth box are in horizontal position. To address this problem, DIoU^37^ improved the convergence speed of the loss function by taking the distance of the line between the center points of the prediction box and the ground truth box into the loss calculation on the basis of IoU. Subsequently. CIoU^38^ made an improvement on DIoU by introducing the aspect ratio into the loss calculation, which further improved the convergence speed of the loss function. However, when the case of the same aspect ratio between the prediction box and the ground truth box is encountered, the penalty term of the aspect ratio of the CIoU loss function is constant to zero and the fluctuation of the convergence process is relatively large. Therefore, in order to make the loss function converge more and quickly, it is decided to make a more precise representation of the loss function in this paper.

## Improved YOLOv7 network

The pipeline weld surface defect detection model in this paper is shown in Fig. 1. The main structure of YOLOv7 model is circled by the red rectangle, and the numbers near each small module indicate the size and number of channels when the feature map passes through the module. The black rectangle is circled by the more detailed structure of some modules in YOLOv7 model. On the basis of YOLOv7 network model, a Le-HorBlock module is designed and added to the fourth CBS module of the backbone network to improve the feature extraction capability of the network. In addition, the CoordAtt mechanism is added to the end of the backbone network to enhance the characterization ability of target features, suppress interference and improve detection accuracy. Ultimately, the convergence speed of the model is accelerated and the detection speed is improved through the optimization of the original network loss function. The improved model can focus more on the valuable contents and locations of the input image. Therefore, the feature information can be extracted effectively and the detection accuracy can be improved.

### Le-HorBlock module

**Figure 2:**
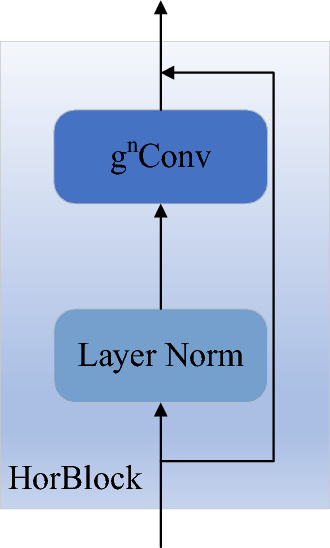
The schematic diagram of HorBlock.

**Figure 3:**
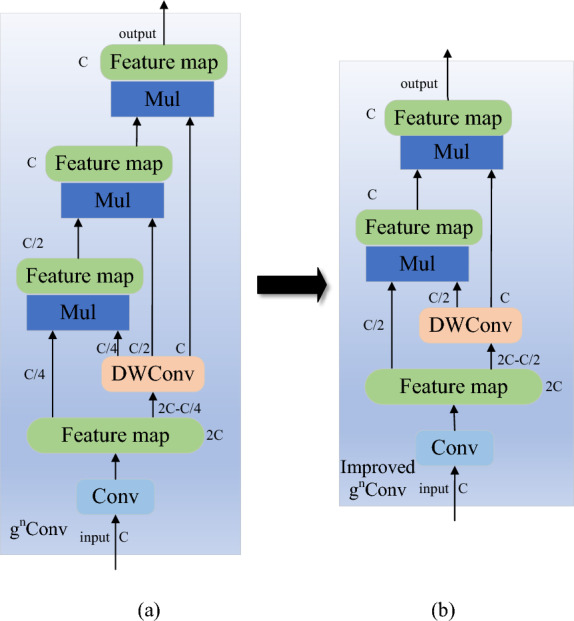
Comparison of g^n^Conv framework before and after improvement: (**a**) g^n^Conv. (**b**) Improved g^n^Conv.

After the network passes through the fourth CBS module of the backbone network, the size of the feature map is reduced to a quarter of the input size, and the feature information is also greatly reduced. Therefore, in order to make the YOLOv7 network extract features more fully, a Le-HorBlock block is added after the fourth CBS block of the backbone network in this paper, the structure of Le-HorBlock is shown in Figs. 2 and 3b. The design of this module is based on HorBlock^39^, and it consists of g^n^Conv recursive gated convolution and layer normalization, as shown in Figs. 2 and 3a.

The g^n^Conv module is formed by standard convolution, linear mapping and element multiplication. During layer normalization, all channels' mean and variance are calculated, then normalized. The main structure of gated convolution is similar to that of standard convolution, except that a gating mechanism is added to its convolution layer. The g^n^Conv module first adjusts the number of feature channels by passing the incoming feature map through two convolutional layers, and then divides the output features of the deeply separable convolution into multiple parts, and each part of the divided part interacts with the previous part in an element-by-element multiplication to finally obtain the output features. Through element multiplication and recursive design, the interactive fusion of high and low order information of feature map is realized, which makes the information contained in feature map more abundant and reduces the phenomenon of gradient diffusion. Thereby enhancing the ability of network feature extraction. The recursion here is to continuously perform the element-by-element multiplication operation.

The detailed implementation of g^n^Conv is shown in the following formula:

Let $x \in R^{HW \times C}$ be the input feature, then the output of gated convolution $y = gConv(x)$ can be expressed as:

1 $$\phi_{in} (x) = [p_{0}^{{}} ,q_{0}^{{}} ],\begin{array}{*{20}c} {} & {} \\ \end{array} \phi_{in} (x) \in R^{HW \times 2C}$$

2 $$p_{1} = f(q_{0} ) \odot p_{0}$$

3 $$y = \phi_{out} (p_{1} )$$

where $\phi_{in}$,$\phi_{out}$ are linear projection layers to perform channel mixing, $f$ is a depth-wise convolution. Therefore, the above formulation introduces the interaction among the neighboring features $p_{0}^{{}}$ and $q_{0}^{{}}$ through element-wise multiplication. The interaction in gConv is regarded as the 1-order interaction, because each $p_{0}$ interacts with its neighboring feature $q_{0}$ only once.

After realizing the 1-order spatial interaction, g^n^Conv is designed by recursive theory. Formally, a set of projection features $p_{0}$ and $\{ q_{k} \}_{k = 0}^{n - 1}$ are obtained by using $\phi_{in}$, which is expressed as:

4 $$\phi_{in} (x) = [p_{0}^{{HW \times C_{0} }} ,q_{0}^{{HW \times C_{0} }} , \cdots ,q_{n - 1}^{{HW \times C_{n - 1} }} ]$$

Then recursively perform gated convolution:

5 $$p_{k + 1} = f_{k} (q_{k} ) \odot g_{k} (p_{k} )/\alpha ,\begin{array}{*{20}c} {} & {} \\ \end{array} k = 0,1, \cdots ,n - 1$$

where the output is scaled by $1/\alpha$ to stabilize the training, $\{ f_{k} \}$ are a set of depth-wise convolution layers, $\{ g_{k} \}$ are used to match dimension in different orders, and $\{ C_{k} \}$ are used to calculate the channel dimension divided each time.

6 $$g_{k} = \left\{ {\begin{array}{*{20}c} {Identity,} \\ {Linear(C_{k - 1} ,C_{k} ),} \\ \end{array} } \right.\begin{array}{*{20}c} {k = 0,} \\ {\begin{array}{*{20}c} {} & {} \\ \end{array} 1 \le k \le n - 1.} \\ \end{array}$$

7 $$C_{k} = \frac{C}{{2^{n - k - 1} }},\begin{array}{*{20}c} {} & {} \\ \end{array} 0 \le k \le n - 1$$

The output of the last recursive $q_{n}$ is sent to the projection layer $\phi_{out}$, and the result of g^n^Conv is obtained. From Eq. (5), it can be seen that the interaction order of $p_{k}$ will increase by 1 after each step. Therefore, g^n^Conv realizes the n-order spatial interaction.

In Fig. 3, g^n^Conv implements cubic matrix multiplication and is represented by Mul operations, which representing the interaction of third-order information. Therefore, in order to explore the effect of gnConv order on the weld defect detection model, the gnConv order n is set as 2, 3 and 4 respectively, and conducts three sets of experiments. The experimental results are shown in Table 1.

**Table 1:** Comparison of experimental results in three cases.

n	mAP@0.5	FLOPS (G)	Average detection speed (s/image)
2	0.729	107.5	0.016
3	0.728	110.2	0.021
4	0.729	115.4	0.032

As can be seen from the results in Table 1, when the order is set to 2, the FLOPS of the model is 107.5, and the detection speed is 16 ms/image, which is superior to the other two schemes, and the detection speed and FLOPS are significantly improved without affecting the detection accuracy. Therefore, the order n of g^n^Conv is set to 2 in this paper, and a new Le-HorBlock module is designed and added to the YOLOv7 model, which greatly enhances the feature extraction ability of the model, although the calculation cost is slightly increased. Through the fusion of high-order feature information and low-order feature information, the weld defect feature map extracted by YOLOv7 model is greatly enriched.

### CoordAtt mechanism

**Figure 4:**
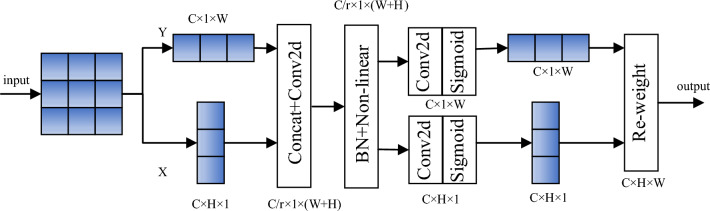
The schematic diagram of CoordAtt mechanism.

In order to further improve the extraction of weld defect features on pipeline surface by YOLOv7 model and suppress useless features, the CoordAtt mechanism^40^ is introduced into the model, which considers the relationship between location information and inter-channel at the same time. It not only captures cross-channel information, but also captures direction-aware and position-aware information, which enables the model to locate and identify the target area more accurately. The schematic diagram of CoordAtt mechanism is shown in Fig. 4, and its operation process is mainly divided into two steps, which are coordinate information embedding and coordinate attention generation.

1. Coordinate information embedding
  The global encoding of channel attention information is usually done using global pooling, but the global spatial information is compressed into the channel descriptors leading to difficulties in preserving the location information. To motivate the attention module to capture remote spatial interactions with precise location information, the global pooling is decomposed into a pair of one-dimensional feature encoding operations. Specifically, given the input X, each channel is first encoded along the horizontal and vertical coordinates using a pooling kernel of size (H, 1) or (1, W), respectively. Thus, the output of the c-th channel with height h can be expressed as:
  8 $$z_{c}^{h} (h) = \frac{1}{W}\sum\limits_{0 \le i < W} {x_{c} } (h,i)$$
  similarly, the output of the c-th channel with width w can be written as:
  9 $$z_{c}^{w} (w) = \frac{1}{H}\sum\limits_{0 \le j < H} {x_{c} (j,w)}$$
  A pair of direction-aware feature maps is obtained by the above two transformation operations. This operation corresponds to the X-direction operation and the Y-direction operation in Fig. 4.
2. Coordinate attention generation
  Coordinate attention generation is mainly to concatenate the two previously generated feature maps ${\text z}^{h}$ and ${\text z}^{w}$, and then pass them into the shared 1 × 1 convolution transformation function $F_{1}$. The generated $f$ is an intermediate feature map that encodes spatial information in the horizontal and vertical directions, as shown in Eq. (10).
  10 $$f = \delta (F_{1} ([{\text z}^{h} ,{\text z}^{w} ]))$$
  where [ , ] denotes the concatenation operation along the spatial dimension and $\delta$ denotes the nonlinear activation function. Then decompose $f$ into two independent tensors $f^{h} \in R^{C/r \times H}$ and $f^{w} \in R^{C/r \times W}$ along the spatial dimension, and then two 1 × 1 convolutions $F_{h}$ and $F_{w}$ are used to transform the feature maps $f^{h}$ and $f^{w}$ into the same channel as the input X. The formula is expressed as follows:
  11 $$g^{h} = \sigma (F_{h} (f^{h} ))$$
  12 $$g^{w} = \sigma (F_{w} (f^{w} ))$$
  Where $\sigma$ denotes the sigmoid function. Finally, $g^{h}$ and $g^{w}$ are used as attention weights, while the output of CoordAtt can be expressed as Formula (13).
  13 $$y_{c} (i,j) = x_{c} (i,j) \times g_{c}^{h} (i) \times g_{c}^{w} (j)$$
  Through the above process, the CoordAtt mechanism achieves attention in both horizontal and vertical directions, an approach that has the advantage of capturing long-range correlations along one spatial direction and maintaining precise position information along the other. Its final generated pair of orientation-sensitive and position-sensitive feature maps is complementarily applied to the input feature maps to enhance the representation of the object of interest.
  In order to further explore where the CoordAtt mechanism is placed on the network to maximize the detection effect, experiments are carried out in the following three situations, and the experimental results are shown in Table 2.

**Table 2:** Comparison of experimental results in three cases.

Strategy	mAP@0.5	FLOPS(G)
A	0.728	106.42
B	0.715	107.5
C	0.721	109.1

A. Add the CoordAtt mechanism only at the end of the backbone network
B. Add the CoordAtt mechanism only at the end of the head network
C. Add the CoordAtt mechanism to both the backbone network and the head network.

As can be seen from the experimental results in Table 2, the network performance is the best when only CoordAtt mechanism is added to the backbone network, and the performance is improved compared with the original network.

### Optimization of loss function

The loss function of the YOLOv7 model consists of three parts: localization loss ($L_{loc}$), confidence loss ($L_{conf}$), and classification loss ($L_{cls}$). The total loss is the weighted sum of the above three losses and is calculated as shown in Eq. (14).

14 $$LOSS = W_{1} \times L_{{{\text{loc}}}} + W_{2} \times L_{{{\text{conf}}}} + W_{3} \times L_{{{\text{cls}}}}$$

where $W_{1}$, $W_{2}$ and $W_{3}$ are the weight values of the three loss functions, respectively.

In the specific calculation of the loss function, both confidence loss and classification loss are calculated using the binary cross-entropy loss function, while the localization loss is calculated using the CIoU loss function as the formula shown in (15).

15 $$LOSS_{CIoU} = 1 - IoU + \frac{{\rho^{2} (b,b^{gt} )}}{{c^{2} }} + \alpha v$$

16 $$v = \frac{4}{{\pi^{2} }}(\arctan \frac{{w^{gt} }}{{h^{gt} }} - \arctan \frac{w}{h})^{2}$$

17 $$\alpha = \frac{v}{(1 - IoU) + v}$$

18 $$IoU = \frac{A \cap B}{{A \cup B}}$$

where $\rho^{2} (b,b^{gt} )$ represents the Euclidean distance between the center point of the prediction box and the ground truth box, c represents the diagonal distance of the smallest rectangle that can cover both the prediction box and the ground truth box, $w^{gt}$ and $h^{gt}$ represent the width and height of the ground truth box, w and h represent the width and height of the prediction box, respectively, v is the parameter used to describe the proportional consistency of the aspect ratio of the prediction box and the ground truth box, α is the parameter used to balance the ratio , IoU represents the intersection ratio of the prediction box and the ground truth box.

From the definition of Eq. (16), when the aspect ratio of the prediction box is as large as that of the ground truth box, v = 0. At this time, the penalty term of the aspect ratio does not play a role and the CIoU loss function does not get a stable expression. In addition, the traditional loss functions such as GIoU, DIoU, and CIoU, all of these loss functions only consider the distance, overlap area and aspect ratio between the prediction box and the real ground box during the operation, and ignore the angular relationship between the prediction frame and the real frame, which leads to a slow convergence speed. Therefore, the SIoU loss function^41^ is used to replace the original network CIoU loss function in this paper. The total degrees of freedom of the loss function are reduced and the training convergence of the model is accelerated by redefining the penalty measure by including the angle cost in the loss regression calculation.

**Figure 5:**
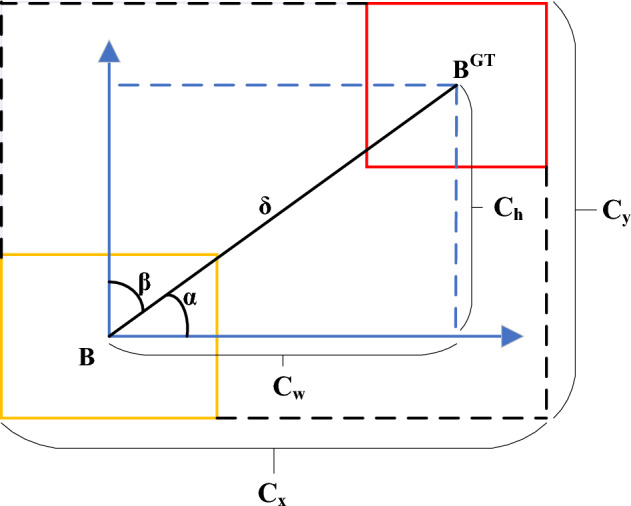
The calculation schematic diagram of SIoU loss function.

The SIoU loss function consists of four components: Angle cost, Distance cost, Shape cost, and IoU cost. The schematic diagram of the SIoU loss function calculation is shown in Fig. 5.

1. Angle cost
  In the calculation of the angle cost, it is first determined whether α or β minimization is used as a rubric by judging whether the angle is greater than 45°, and the angle cost is calculated as shown in Eq. (19).
  19 $$\Lambda = 1 - 2 * \sin^{2} (\arcsin (x) - \frac{\pi }{4})$$
  20 $$x = \frac{{c_{h} }}{\delta } = \sin \alpha$$
  21 $$\delta = \sqrt {{\text{(b}}_{{c_{x} }}^{gt} - b_{{c_{x} }} )^{2} + (b_{{c_{y} }}^{gt} - b_{{c_{y} }} )^{2} }$$
  22 $$c_{h} = \max (b_{{c_{y} }}^{gt} ,b_{{c_{y} }} ) - \min (b_{{c_{y} }}^{gt} ,b_{{c_{y} }} )$$
  among them, $(b_{{c_{x} }}^{gt} ,b_{{c_{y} }}^{gt} )$ represents the coordinates of the center point of the ground truth box; $(b_{{c_{x} }}^{{}} ,b_{{c_{y} }}^{{}} )$ represents the coordinates of the center point of the prediction box, and other symbols are represented in Fig. 5.
2. Distance cost
  The distance cost represents the distance between the center point of the ground truth box and the prediction box. Combined with the angle cost defined above, SIoU defines the distance cost in the following Eq. (23).
  23 $$\Delta = \sum\limits_{{{\text{t}} = x,y}} {(1 - e^{{ - \gamma \rho_{t} }} )}$$
  24 $$\rho_{x} = (\frac{{b_{cx}^{gt} - b_{cx} }}{{c_{w} }})^{2}$$
  25 $$\rho_{{\text{y}}} = (\frac{{b_{cy}^{gt} - b_{cy} }}{{c_{h} }})^{2}$$
  26 $$\gamma = 2 - \Lambda$$
3. Shape cost
  The shape cost is defined as shown in Eq. (27).
  27 $$\Omega = (1 - e^{{ - \frac{{\left| {w - w^{gt} } \right|}}{{\max (w,w^{gt} )}}}} )^{\theta } + (1 - e^{{ - \frac{{\left| {h - h^{gt} } \right|}}{{\max (h,h^{gt} )}}}} )^{\theta }$$
  Where $\theta$ is an adjustable variable representing the weight of the network on the shape cost, which is set to 1.

In summary, the final calculation formula of the SIoU loss function is shown in Eq. (28):

28 $$LOSS_{SIoU} = 1 - IoU + \frac{\Delta + \Omega }{2}$$

The angle cost is included in the loss calculation, mainly for the calculation of the distance loss between the ground truth box and the prediction box. Usually, in the early stage of model training, the prediction box does not intersect with the ground truth box. Adding the angle cost can accelerate the convergence speed of the distance between the two boxes. And the traditional CIoU loss function converges to the overall shape of the ground truth box and the prediction box, while the SIoU regression loss function converges to the edge of the two boxes to achieve the effect of overall shape convergence.

## Experiments and discussion

### Dataset preparation and preprocessing

#### Image acquisition

**Figure 6:**
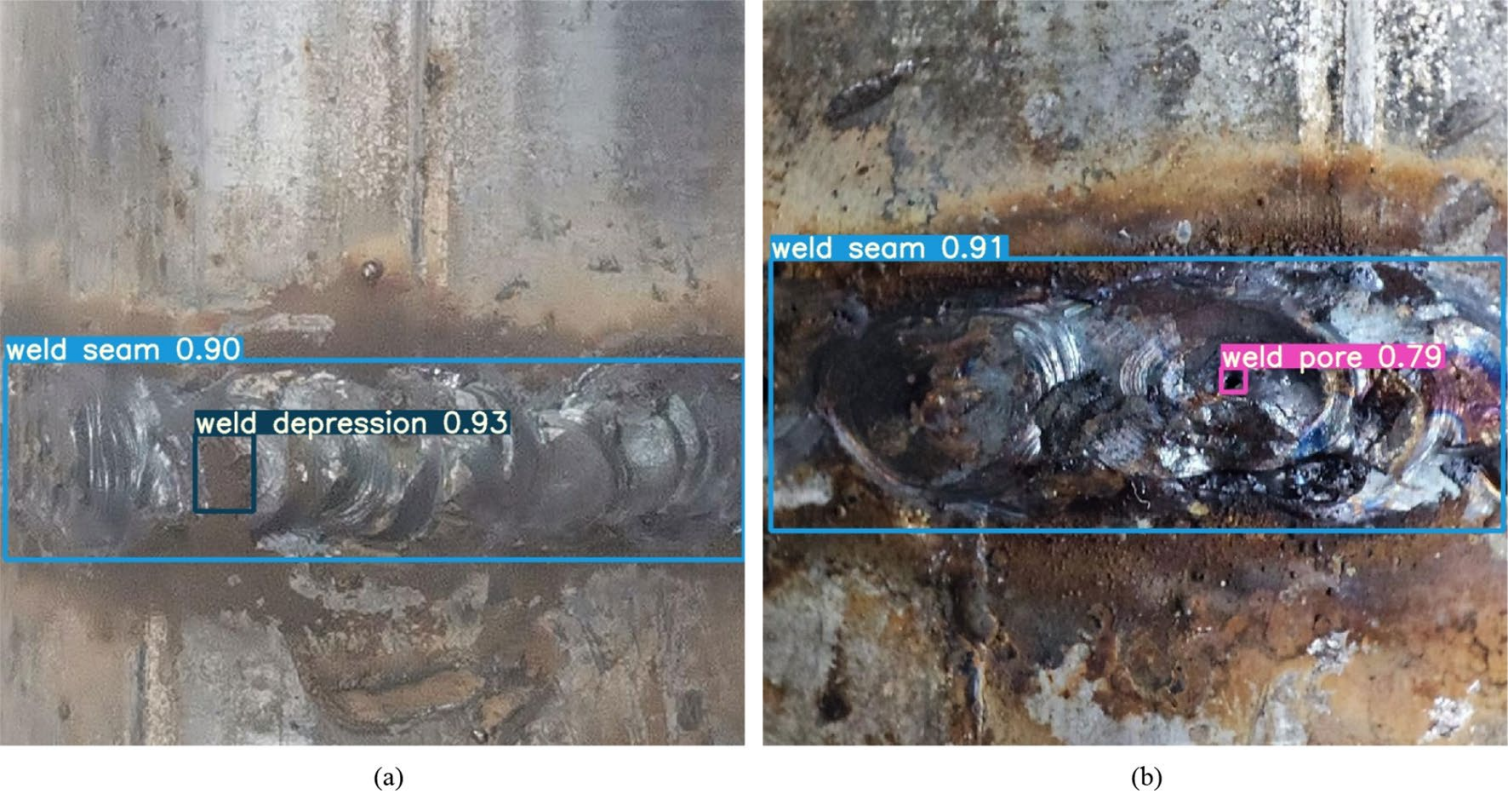
Two different types of defects: (**a**) weld seam in blue boxes and weld depression in cyan boxes, (**b)** weld pore in pink boxes.

There are few data sets for pipeline weld surface defects, and even there is no relatively complete and mature data set in the whole field of weld defect detection. The experimental dataset in this article comes from the laboratory and Baidu and Google. A total of 1000 images are collected. The pixel resolution height and width of the images in this dataset are between 800 and 1000. According to the original image, the pipeline weld surface defect data set is constructed, which mainly includes weld pore and weld depression, as shown in Fig. 6.

#### Image preprocessing

**Figure 7:**
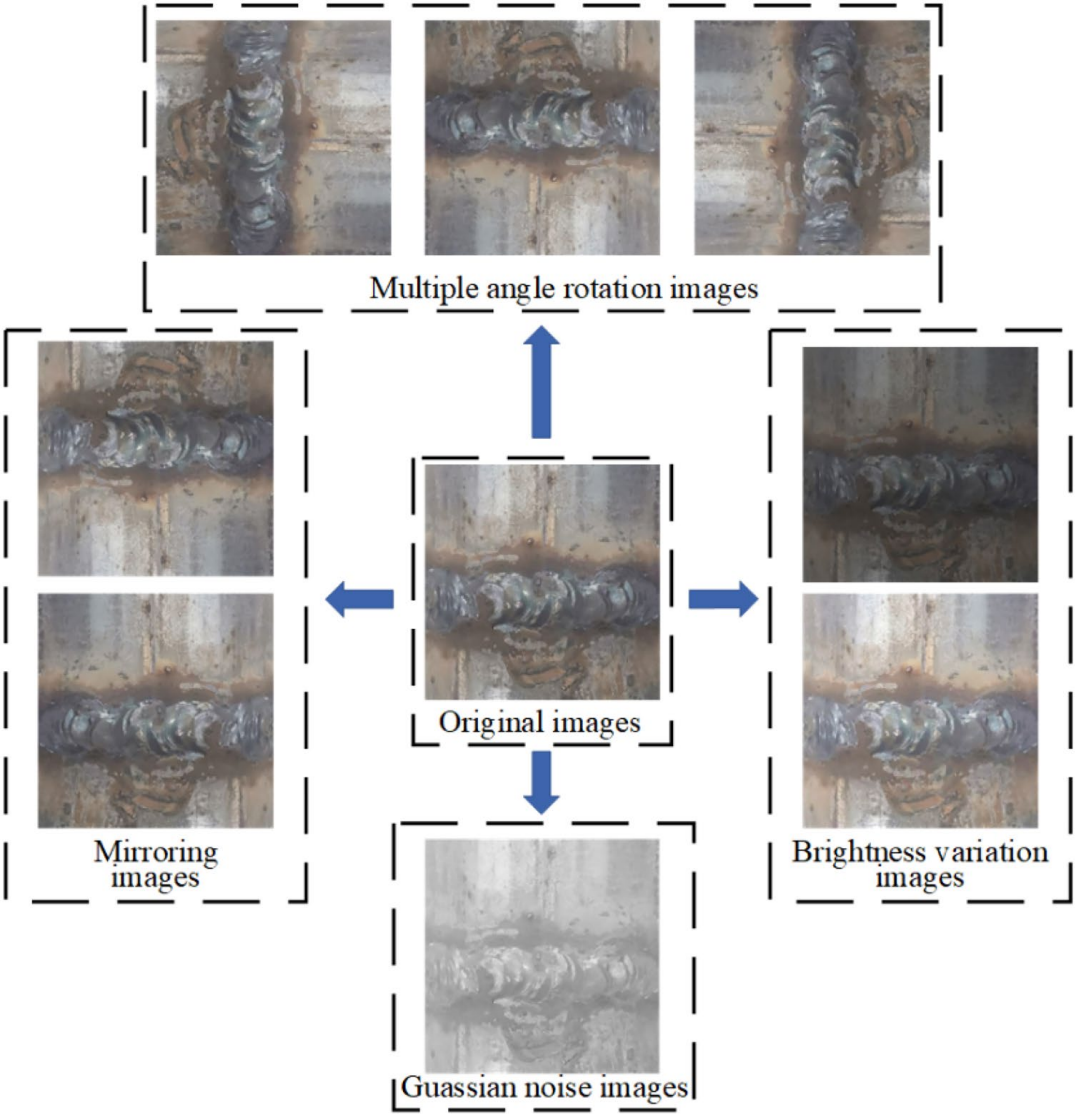
Renderings of data enhancements.

**Figure 8:**
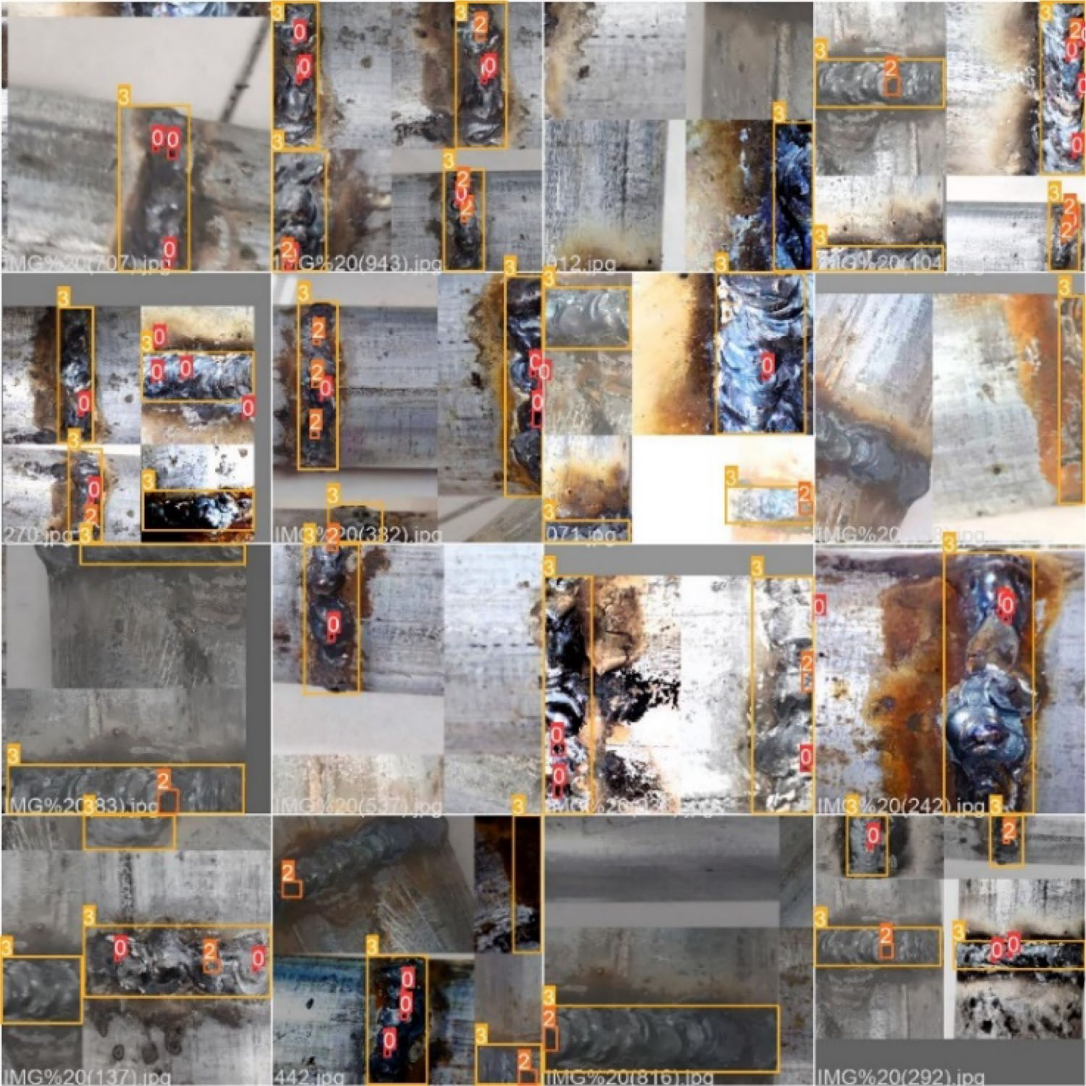
Mosaic data enhancement renderings.

Data samples are required for deep learning models to be trained to solve fitting problems. In the model training stage, the more sufficient and comprehensive the collected data is, the more significant the model recognition effect is^42^. Therefore, the number of samples is expanded by data amplification. The data augmentations strategy used in this paper includes image multi-angle rotation, saturation adjustment image flipping, adding salt and pepper noise, color dithering and other morphological operations, as shown in Fig. 7. Furthermore, the model uses the Mosaic data enhancement method on the input side to improve the classification performance of the model by random scaling, random cropping and random layout stitching of the four defective images. The mixup data augmentation method is used to perform proportional interpolation on two images to realize sample mixing and improve the performance of model classification. After a series of operations, the input of the model is shown in Fig. 8.

#### Image database and label database

**Figure 9:**
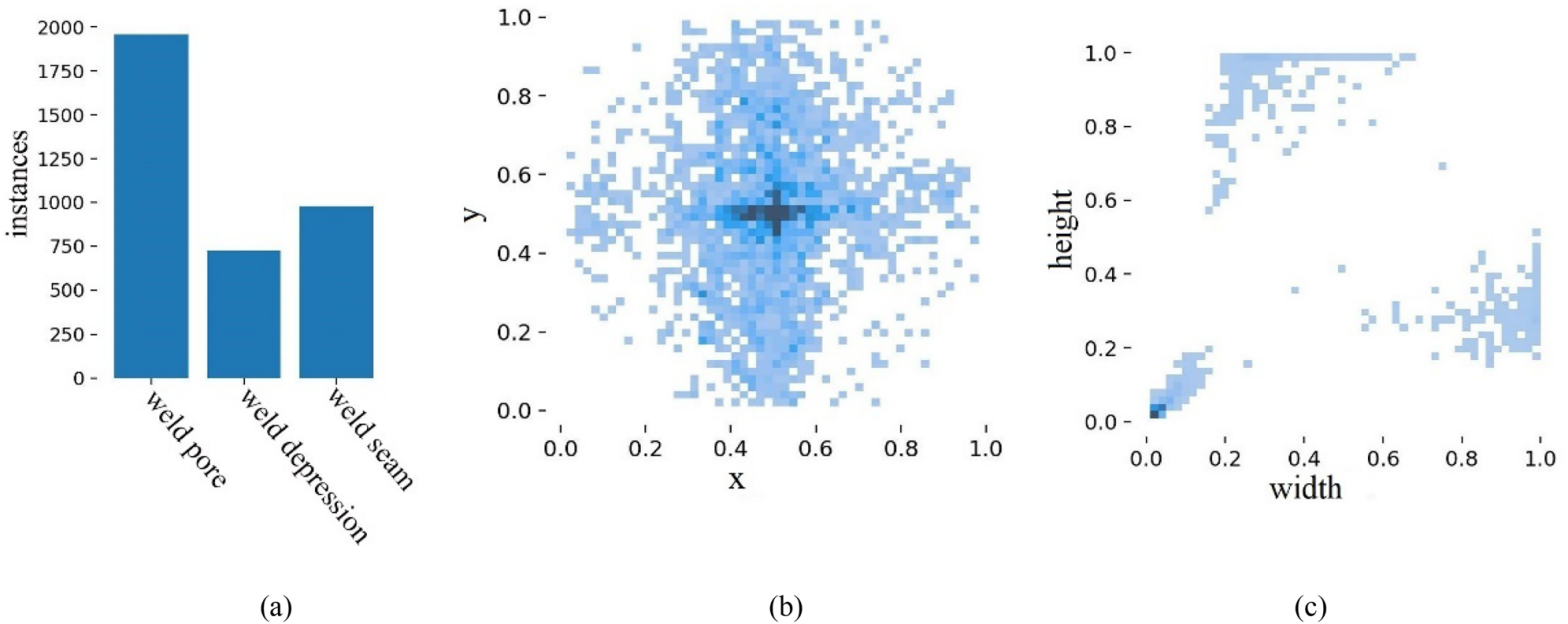
Labels and label distribution: (**a**) number of labels, (**b**) label location, (**c**) label size.

Labelimg tool is used to label the weld and its surface defects in the image. The label categories are weld pore, weld depression and weld seam. A total of 1000 weld images are collected, and the data set is expanded to 2000 by image augmentation. Firstly, according to the previous model training experience^43^, the data set is divided into training set, verification set and test set according to the ratio of 7:2:1, and then the data set is trained according to the ratio of 8:1:1 and 6:3:1 in order to further verify the effectiveness of the improved model. The number and distribution of tags in the statistical data set, and the results are shown in Fig. 9.

Figure 9a shows the number of different labels, with the vertical axis indicating the number of labels and the horizontal axis the name of the label. Sufficient defect samples are available in the dataset to enable inclusion of most surface defect scenarios for pipe welds. Figure 9b shows the distribution of label positions, with the horizontal coordinate x being the ratio of the horizontal coordinate of the center of the label to the width of the image and the vertical coordinate y being the ratio of the vertical coordinate of the center of the label to the height of the image. As can be seen from the figure, the data are widely distributed and concentrated in the middle of the image. In Fig. 9c, the abscissa width is the ratio of the label width to the image width, and the ordinate height is the ratio of the label height to the image height. The dataset contains data of various sizes, with the majority of small-sized target data and a wide range of target sizes.

### Experimental environment and evaluation metrics

The hardware environment and main software configurations used in the experiment are shown in Table 3. The CPU frequency is 2.60 GHz, the GPU is NVIDIA GeForce RTX A5000 (24 GB), and the operating system is Ubuntu 20.04.

**Table 3:** Experimental environment configuration.

Parameter	Configuration
CPU	15vCPU Intel(R) Xeon(R) Platinum 8358P CPU @ 2.60 GHz
GPU	NVIDIA GeForce RTX A5000(24 GB)
Operating system	Ubuntu 20.04
Python	3.8
CUDA	11.3
Torch	1.10.0
Learning rate	0.01
Momentum	0.937
Weight decay	0.0005
Batch size	16
Image size	640 × 640
Epochs	300

In order to comprehensively and objectively evaluate the performance of our model in this paper, a confusion matrix is used for comprehensive evaluation, as shown in Table 4. TP indicates correct detection, that is, the predicted value of the model is positive, and the actual value is also positive; FN represents error detection that is, the predicted value of the model is negative, but the actual value is positive; FP represents error detection, that is, the predicted value of the model is positive, but the actual value is negative. TN represents correct detection, the model prediction is negative, and the actual value is also negative. The expressions for precision and recall (29)(30) are as follows:

29 $$P = \frac{TP}{{TP + FP}}$$

30 $$R = \frac{TP}{{TP + FN}}$$

**Table 4:** Confusion matrix.

Reference\prediction	Positive	Negative
Positive	True positive (TP)	False negative (FN)
Negative	False positive (FP)	True negative (TN)

The average precision (AP) is used as the evaluation index for each defect category, and the mean average precision (mAP) is used to evaluate the performance of the whole network model. The mAP@0.5 (when IOU is set to 0.5, the AP of all images in each category is calculated, and then the average of all categories is calculated) is used as a measure of the performance of the whole model in this paper.

1. AP. Represents the mean value of precision under different recalls. The formula is:
  31 $$AP = \int_{0}^{1} {p(r)dr}$$
2. mAP. Represents the mean of the average precision of all target detection categories, The formula is:
  32 $${\text{m}}AP = \frac{1}{{n_{j} }}\sum\limits_{j = 1}^{{n_{j} }} {Ap_{j} }$$
  Where n is the number of a class; $Ap_{j}$ represents the detection precision of category j.

### Visual analysis of model

**Figure 10:**
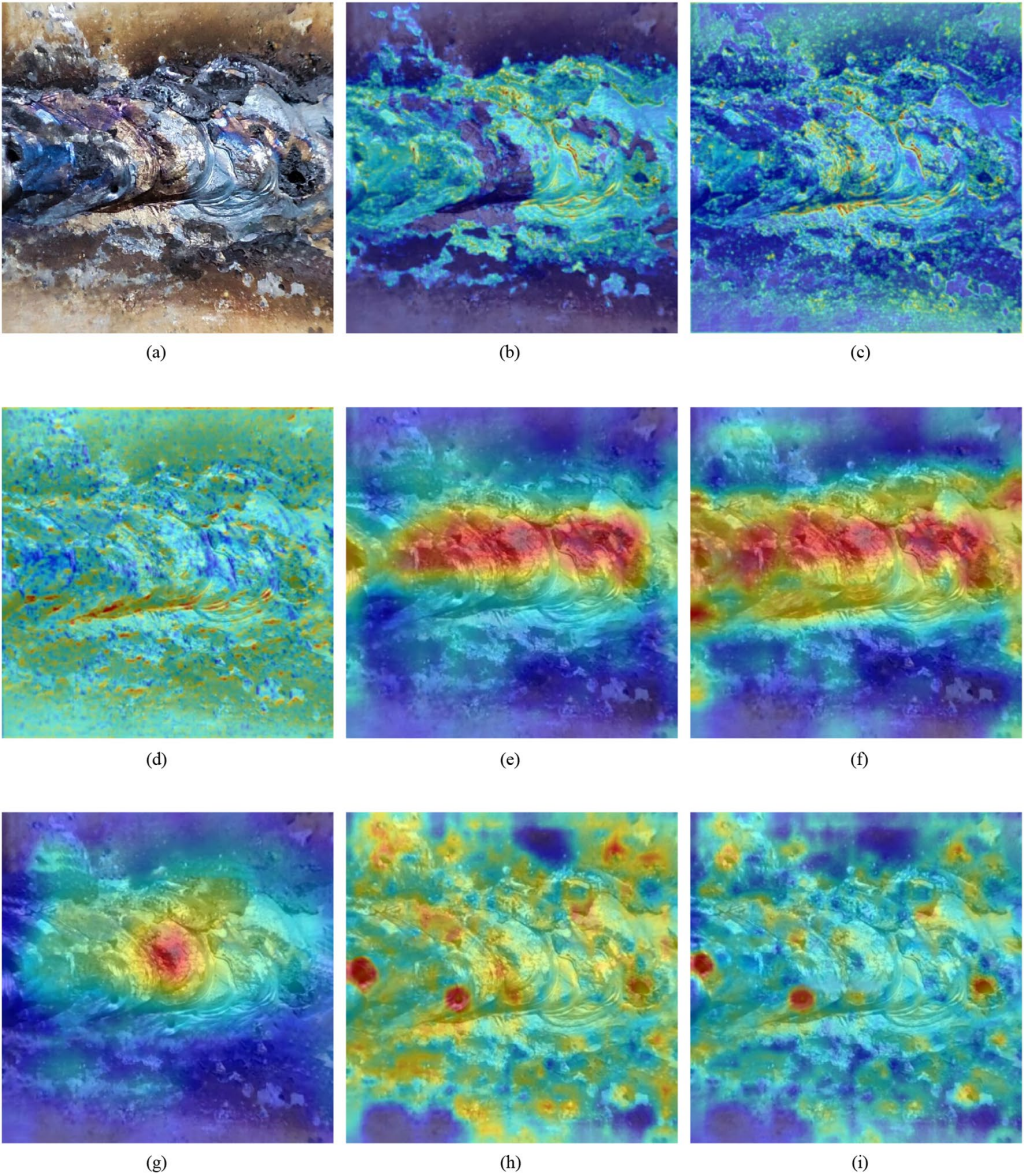
Visualization of the feature map: (**a**) Original image. (**b–d**) Feature maps after the first convolution layer. (**e,f**) Feature maps at the end of backbone. (**g–i**) Feature maps of output of the three detectors.

After training the model, the feature map of the trained model is visualized. The information of interest in the network model can be seen from the visual feature map. Furthermore, it is to check whether the added attention mechanism contributes to the improved model detection. the feature maps of the first convolution module, the output of backbone network module and the output of the three detection heads of the model are visualized. As shown in Fig. 10, which area of the graph is brighter represents the area that the model is more concerned about.

According to the visual feature map after the first convolutional layer, as shown in Fig. 10b, Fig. 10c, d, the extracted features have specific focuses, some on edges and some focus on overall features. In comparison to the deeper features, middle and shallow features are mostly completed, while deeper network features are fewer. After adding the attention mechanism, the feature maps are strengthened, as shown in Fig. 10e, f, and unnecessary features are suppressed, as can be seen in the feature maps output after the backbone network. The feature maps from the last three layers are used to detect large, medium, and small targets, which improves the model's multi-scale detection capability, as shown in Fig. 10g–i.

### Experimental results and analysis

#### Comparison experiment

To verify the effectiveness of the improved method, comparative experiments are conducted in terms of attention mechanism and loss function selection. The baseline of each experiment is the YOLOv7 model. Because the main goal of this paper is to improve the detection accuracy of the model, the experiments use precision, recall, and mAP@0.5 to evaluate the effect.

Different loss functions are chosen to perform multiple sets of experiments. YOLOv7 uses CIoU as its loss function. Therefore, the performances of DIoU, CIoU and SIoU loss function (ours) are compared. The results are shown in Table 5.

**Table 5:** Performances of different loss functions.

Loss function	P	R	mAP@0.5
DIoU	0.933	0.74	0.618
CIoU	0.940	0.73	0.627
SIoU	0.968	0.73	0.706

**Figure 11:**
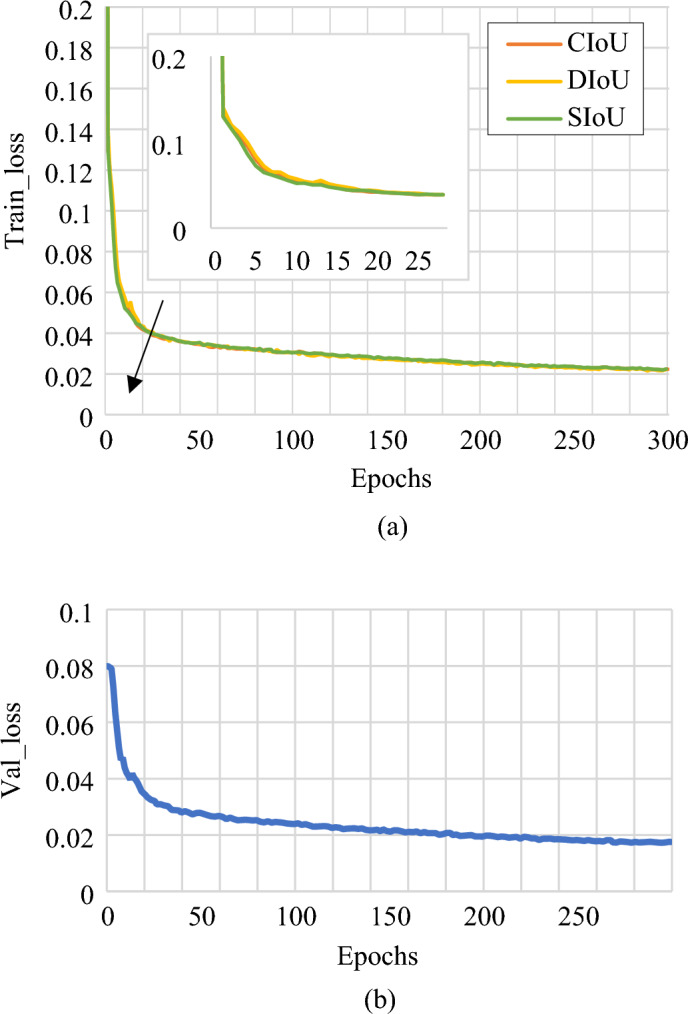
Loss function iteration comparison: (**a**) Train_loss, (**b**) Val_loss.

The experimental results show that the precision of SIoU loss function is about 2.8% higher than that of CIoU, and mAP@0.5 is 8.8% higher. At the same time, by comparing the curves of the training loss and the verification loss of these two loss functions with the number of iterations, the convergence of the models using different loss functions is verified, as shown in Fig. 11. The curves in the figure indicate the convergence of the average bounding box loss when CIoU and SIoU are used for the bounding box loss, respectively.

As can be seen in Fig. 11a, the training loss of the model using CIoU and SIoU loss functions eventually converge as the number of iterations increases, but the SIoU loss function converges more rapidly at around Epochs = 5. Moreover, it is found from Fig. 11b that the verification loss of the model using the SIoU loss function eventually tends to converge, which indicates that the model trained with the improved network structure can fit the verification set data well, and there is no over-fitting or under-fitting. SIoU loss function can be used to detect weld surface defects better with the data set in this paper.

To further verify the effects of different attention mechanism on the model, SE and CBAM is added to the same location in the network respectively for comparison with the CoordAtt mechanisms. The results are shown in Table 6. As can be seen from Table 6, compared with the original model, the mAP@0.5 of YOLOv7 added with SE mechanism is improved by 8.4%, and the detection performance is slightly improved. The YOLOv7 model with CBAM mechanism is 9.3% higher than the original model mAP@0.5, and the overall performance of the model is improved, but not obvious. The YOLOv7 network model with CoordAtt mechanism is 10.1% higher than the original model mAP@0.5, and the calculation amount is also lower than the model with CBAM mechanism, which reduces the calculation pressure of the hardware. The analysis and comparison of the experimental results show that the model with the addition of the CoordAtt mechanism outperforms the original model and the model with the addition of the SE and CBAM mechanisms, which proves that the use of the CoordAtt mechanism allows the network model to pay more attention to the target and improves the detection capability of the network.

**Table 6:** Effects of models with different attention mechanisms.

Strategy	mAP@0.5	FLOPS(G)
YOLOv7	0.627	105.2
YOLOv7 + SE	0.711	106.5
YOLOv7 + CBAM	0.720	106.5
YOLOv7 + CoordAtt(ours)	0.728	106.42

#### Ablation experiment

To verify the effectiveness of the YOLOv7 improvement strategy proposed in this paper, ablation experiments are conducted one by one. The combination experiments are conducted for each improvement strategy separately, and the experimental results are shown in Table 7. "√" indicates that the improvement method is adopted, and " × " indicates that the improvement method is not adopted.

**Table 7:** Ablation experiment results.

Methods	Baseline	Le-HorBlock	CoordAtt	SIoU	P	R	mAP@0.5	FLOPS (G)
A	YOLOv7	×	×	×	0.940	0.73	0.627	105.2
B		√	×	×	0.918	0.95	0.729	107.5
C		√	√	×	0.955	0.91	0.734	108.4
D		×	√	√	0.960	0.81	0.728	106.4
E		√	√	√	0.968	0.96	0.789	109.6

Method (a) in Table 7 is the original YOLOv7 model, and its detection precision and mAP@0.5 reached 0.940 and 0.627. Method (b) is to add the Le-HorBlock module to the original module to the original YOLOv7 model, and the recall of the model is improved obviously, which is 22% higher than that of method (a), which shows that it can obviously improve the phenomenon of missed detection. And mAP@0.5 increased by 10.2%. On the basis of slightly increasing the computing cost, the module improved recall and mAP@0.5, and effectively improved the detection performance.

On the basis of method (b), the CoordAtt mechanism is added to method (c). The CoordAtt mechanism improves the feature extraction capability of the network, enabling it to capture more accurate location information and target features and suppress the influence of interference factors on the detection results. From the experimental results, the inclusion of the CoordAtt mechanism improves the precision by 3.7% compared with method (b), which can effectively improve the detection accuracy with little change in computational volume. Method (d) based on method (a), CoordAtt mechanism is added and loss function is optimized. From the experimental results in Table 7, it can be concluded that the accuracy of the model increased by 2% after adding the above two improvement points. Compared with the experimental results only adding attention mechanism, the loss function is optimized on this basis, which further improved the detection effect of the model.

Method (e) optimizes the loss function based on method (c), and after replacing the loss function with SIoU, the precision increases by 1.3% and mAP@0.5 by 5.5% compared with method (c). Based on the above three groups of improved methods, it can be found that the combined improved algorithm has the best effect, the detection accuracy can reach 96.8%, and the mAP@0.5 reaches 78.9%. It has the characteristics of high precision and low leakage rate, and can meet the requirements of pipeline weld surface defect detection.

Table 8. shows the detection results for each category using different detection models. From the experimental results, the improved YOLOv7 network model proposed in this paper improves the detection precision of weld pore defects by 7.3% and the detection of weld depression defects by 41.3% on average compared to the original model, which shows its superiority in detecting targets in complex background and small size targets. In addition, the large difference in detection precision of the improved YOLOv7 network model for the three categories of weld pore, weld depression, and weld seam is due to the complex characterization environment and tiny targets of pore and depression defects, so their precision and recall are not as good as those of welds.

**Table 8:** Detection effect of the model on each category.

Class	AP				
	Faster R-CNN	YOLOv3	YOLOv5	YOLOv7	Our model
Weld pore	0.628	0.688	0.714	0.721	0.794
Weld depression	0.161	0.153	0.158	0.167	0.580
Weld seam	0.972	0.991	0.992	0.995	0.994

In order to make the improved model more convincing, three different ways of dividing data sets are used to train the model, and finally the average value is taken to measure the detection effect of the model. Experiments are carried out on the data set according to the ratio of 6:3:1, 7:2:1 and 8:1:1, respectively. The experimental results are shown in Table 9.

**Table 9:** Detection effect of models trained with different data set division ratios.

Divide proportion	P	R	mAP@0.5
6:3:1	0.960	0.91	0.782
7:2:1	0.968	0.96	0.789
8:1:1	0.966	0.97	0.788
Average value	0.965	0.95	0.786

From the experimental data in Table 9, it can be seen that the final detection effect of the models trained by three different ways of dividing data sets is not much different, and the difference of mAP@0.5 is kept within 1%. Therefore, the average of the model results trained by three different partition methods is used to represent the effect of the improved model.

**Figure 12:**
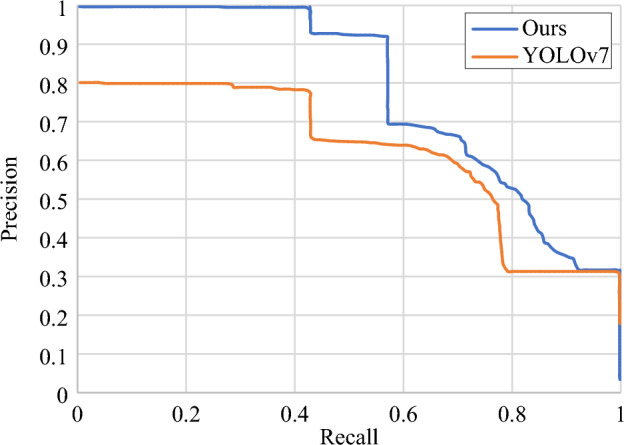
Comparison of PR curves before and after improvement.

Neither precision nor recall can be used as the sole metrics for assessing model performance. Therefore, the PR curve is chosen for further evaluation of the model since it not only records the detection performance of the whole model, but also records the detection effect of the model for each type of defect separately. The PR curves of the original YOLOv7 network model and the proposed network model are shown in Fig. 12. Its horizontal axis represents recall, vertical axis represents precision. Figure 12 shows the situation that the precision changes with the recall rate intuitively. If the curve in the figure is close to the upper right corner, it means that with the improvement of the recall, the decline in precision is not obvious, the overall performance of the model is better. It can be seen that the curve of our model is closer to the upper right corner than the original model.

#### Comparison experiments with other models

**Figure 13:**
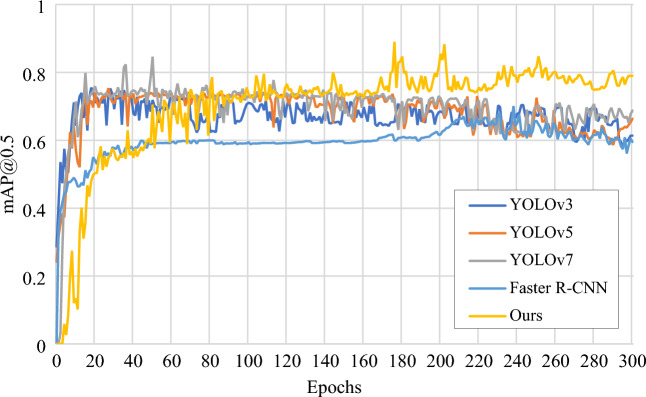
mAP@0.5 curves of each model.

The ablation experiment can only prove that the improved algorithm is effective compared with the original algorithm, but whether it can reach the advanced level remains to be proved. Therefore, the improved YOLOv7 network model is compared with other classical target detection models to verify the effectiveness of the improved algorithm under the condition that the configuration environment and initial training parameters are consistent. Figure 13 shows the mAP@0.5 curves of YOLOv7, three other target detection algorithms and the algorithm in this paper, and it can be seen from the figure that the mAP@0.5 of the improved algorithm is significantly higher than that of the other four models. The experimental results are shown in Table 10, the improved YOLOv7 network model in the case of input of the same size of the picture, compared with the original YOLOv7 network model, the precision increased by 2.8%, the recall increased by 22%, the mAP@0.5 increased by 15.9%, and surpassed all other network models.

**Table 10:** Comparison of results between improved YOLOv7 model and other models.

Model	Input Size	P	R	mAP@0.5	Average detection speed (s/image)
Faster R-CNN	640 × 640	0.78	0.64	0.587	0.035
YOLOv3	640 × 640	0.75	0.72	0.611	0.018
YOLOv5	640 × 640	0.897	0.91	0.621	0.012
YOLOv7	640 × 640	0.94	0.73	0.627	0.011
Our model	640 × 640	0.965	0.95	0.786	0.013

**Figure 14:**
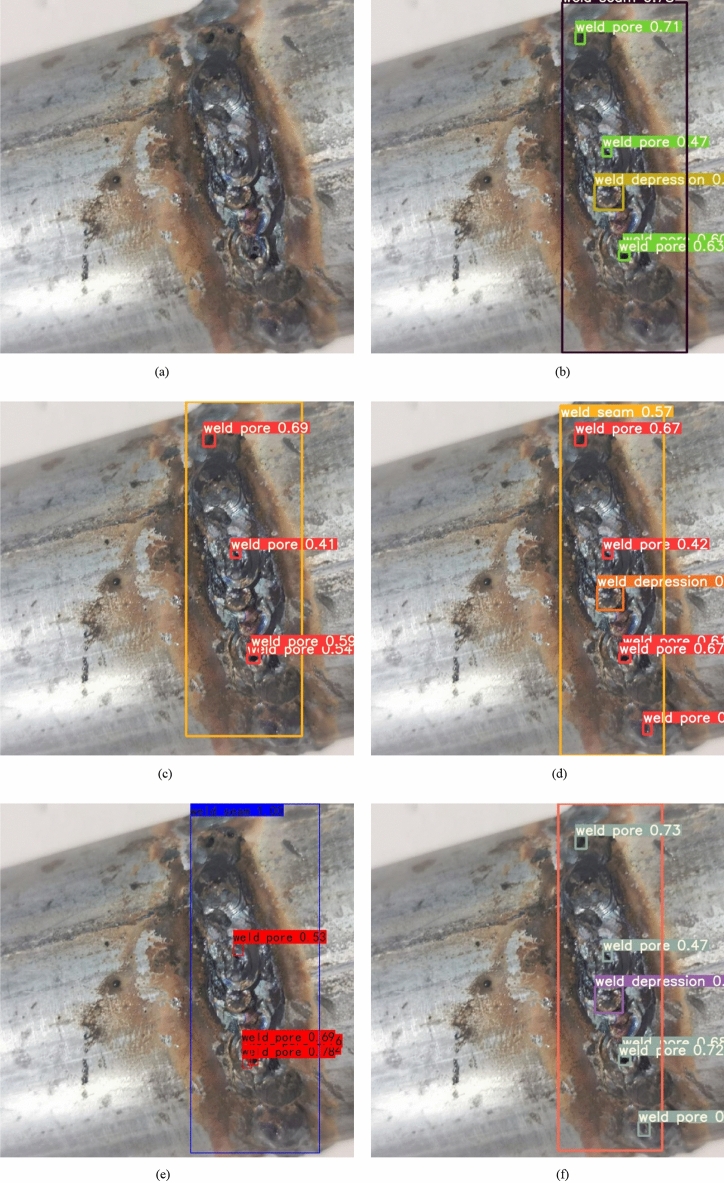
Detection effect of different network models: (**a**) original images, (**b**) YOLOv7, (**c**) YOLOv3, (**d**) YOLOv5, (**e**) faster R-CNN, (**f**) our model.

Through the comparison and analysis of a series of experiments, it can be concluded that the improved YOLOv7 network model proposed in this paper has obvious advantages in detection precision and recall. To verify the model's generalization ability and robustness, targets with complex background are specifically selected for testing. The results are shown in Fig. 14. Through the detection results, it is found that the detection results of YOLOv3, YOLOv7 and Faster R-CNN models have different number of missed detection conditions, while YOLOv5, although all defects in the graph are detected, the confidence level is not as good as the model proposed in this paper. The improved YOLOv7 network model can better identify defects of pipeline weld surface, and can identify some defect targets in complex background.

## Conclusions

The improved YOLOv7 pipeline weld surface defect detection model is proposed. The experimental results show that the model can accurately identify pipeline weld and its surface defects in difficult pipeline weld defect detection tasks. The following conclusions can be drawn from the above research.

1. A Le-HorBlock module is designed and added to YOLOv7 network. By implementing second-order spatial interaction and enhancing the backbone network, the features of weld seam images are extracted, and the feature mapping of weld seam surface defect targets is optimized.
2. By adding the CoordAtt mechanism at the end of the YOLOv7 backbone network, the representation ability of target features is improved, interference is suppressed, and detection accuracy is improved.
3. The optimization of IoU loss function and the use of SIoU loss function instead of the original model loss function have accelerated the introduction of corner cost in loss calculation to accelerate the convergence of the model.

The large dataset of pipeline weld surface defects is prepared from the collected weld images. Comparative and ablation experiments are conducted using this dataset. The test results show that the improved YOLOv7 network can increase the recall by 22% and mAP@0.5 by 15.9%, respectively, compared with the original network, the detection effect is better than the original YOLOv7 network and other classical target detection networks.

## Data Availability

The dataset generated and analyzed during the current research period cannot be made public due to the involvement of many ongoing scientific research projects in the weld image dataset, but can be obtained from corresponding authors according to reasonable requirements.
